# The best from East and West? Acupuncture and medical training therapy as monotherapies or in combination for adult patients with episodic and chronic tension-type headache: study protocol for a randomized controlled trial

**DOI:** 10.1186/s13063-019-3700-1

**Published:** 2019-11-08

**Authors:** J. Schiller, T. Kellner, J. Briest, K. Hoepner, A. Woyciechowski, A. Ostermann, C. Korallus, C. Sturm, T. Weiberlenn, L. Jiang, C. Egen, F. Beissner, M. Stiesch, M. Karst, C. Gutenbrunner, M. G. Fink

**Affiliations:** 1 Department of Rehabilitation Medicine, Hannover Medical School, Hannover, Carl-Neuberg-Straße 1, 30625 Germany; 2 Medical practice for Traditional Chinese Medicine, Hannover, Germany; 3 Somatosensory and Autonomic Therapy Research, Institute for Neuroradiology, Hannover Medical School, Hannover, Germany; 4 Department of Prosthetic Dentistry and Biomedical Materials Science, Hannover Medical School, Hannover, Germany; 5 Department of Anesthesiology and Intensive Care Medicine, Hannover Medical School, Hannover, Germany

**Keywords:** Acupuncture, Episodic tension-type headache, Chronic tension-type headache, Medical training therapy, Exercise, Study protocol, Randomized controlled trial, Traditional Chinese Medicine

## Abstract

**Background:**

This study aims to evaluate the feasibility and efficacy of a complex health intervention, based on the combination of conventional Western medicine and traditional Chinese medicine (TCM), in an outpatient department of a university hospital for patients with frequent episodic or chronic tension-type headaches.

**Methods/design:**

This is a prospective randomized controlled pilot study with four balanced treatment arms (usual care, acupuncture, training, and training plus acupuncture). Each arm will have 24 patients. After the initial screening examination and randomization, a 6-week treatment period follows, with treatment frequencies decreasing at 2-week intervals. After completion of the intervention, two follow-up evaluations will be performed 3 and 6 months after the start of treatment. At predefined times, the various outcomes (pain intensity, health-related quality of life, pain duration, autonomic regulation, and heart rate variability) as well as the participants’ acceptance of the complex treatment will be evaluated with valid assessment instruments (Migraine Disability Assessment, PHQ-D, GAD-7, and SF-12) and a headache diary. The acupuncture treatment will be based on the rules of TCM, comprising a standardized combination of acupuncture points and additional points selected according to individual pain localization. The training therapy comprises a combination of strength training, endurance training, and training to improve flexibility and coordination. Besides descriptive analyses of the samples, their comparability will be assessed using an analysis of variance (ANOVA) or chi-squared tests. Analyses will be performed on an intention-to-treat basis. Potential interaction effects will be calculated using a repeated-measures ANOVA to test the primary and secondary hypotheses. In supplementary analyses, the proportion of treatment responders (those with a 50% reduction in the frequency of pain episodes) will be determined for each treatment arm.

**Discussion:**

This trial may provide evidence for the additive effects of acupuncture and medical training therapy as a combination treatment and may scientifically support the implementation of this complex health intervention.

**Trial registration:**

Registered on 11 Feburary 2019. German Clinical Trials Register, DRKS00016723.

## Background

Both acupuncture and medical training therapy (MTT) are used as monotherapies to treat tension-type headaches. The usefulness of both acupuncture and MTT as effective treatment options with moderate but clinically relevant effect sizes has been demonstrated in meta-analyses [1, 2]. The benefits of acupuncture treatment have been shown in studies comparing acupuncture with usual care [3, 4] and so-called sham acupuncture [5]. For MTT, significant reductions in the frequency of headache attacks [6] and in analgesic consumption [7] have been demonstrated in patients with tension-type headaches. Compared with acupuncture, MTT has been shown to significantly improve quality of life and patient satisfaction [8, 9]. Despite the evidence in support of the effectiveness of acupuncture and MTT, we could not identify any studies evaluating the efficacy of a combination of acupuncture and MTT for tension-type headaches in comparison with the two treatment modalities alone or with usual care.

Tension-type headaches are common. They have a significant socioeconomic impact, primarily due to disease-associated incapacity for work and medical treatment costs [10, 11]. Whether complementary combination therapies are more effective than each treatment modality alone is of clinical relevance and has not yet been answered. In this study, acupuncture as a treatment modality in traditional Chinese medicine (TCM) is combined with MTT, a classical Western medical treatment.

In addition to the classical outcomes of headache studies, which will be used to evaluate the efficacy of the two methods regarding pain, analgesic consumption, anxiety, and depression, this study will also address whether the two therapies affect individual psychological stress levels, as an association between increased stress levels and tension-type headaches has been reported. Several studies assessing techniques involving physical contact, such as Thai massage, showed positive changes in heart rate variability (HRV), which is used as an objective measure of psychological stress.

In addition, the study will assess whether those participating in the study have a functional disorder of the chewing system (temporomandibular disorder or TMD). Speciali and Dach [12] considered several epidemiological studies in a systematic review, which showed the co-occurrence of TMD and tension-type headaches. Moreover, published studies have shown improvements in headaches when a co-existing TMD was treated with dental interventions alone [13].

By involving experts in TCM, the proposed study may provide information about another interesting aspect, which is that tension-type headaches are described as a mild to moderate, holocranial headaches with a dull, pressing character [14]. However, differences in the location of maximum pain have no impact on treatment decisions based on the International Classification of Headache Disorders (3rd Edition) [15] of the International Headache Society (IHS). By contrast, the acupuncture points to be used for treatment are selected according to the meridian diagnosis of TCM, which is primarily guided by where the pain is located and where it radiates to. Independent of investigating whether selecting acupuncture points according to TCM criteria is superior to the unspecific effect of random needling, the proposed study will investigate the inter-rater reliability of meridian diagnosis. Moreover, the subjective diagnosis of a TCM specialist with an assignment to corresponding meridians will be compared with the patient-created pain mapping with an assignment to local meridians. However, this study will not compare acupuncture with random acupuncture because this would tend to underestimate the effects of acupuncture [16]. As mentioned in the conclusion of the meta-analysis by Linde et al. [1], a non-inert effective therapy (i.e., MTT) will be compared with acupuncture and usual care.

### Research questions

Primary null hypothesis:

- For the primary endpoint, (1) acupuncture and MTT, alone or in combination, are not superior to usual care according to IHS criteria, and (2) the combination of the two non-pharmacological treatment modalities is not superior to either treatment modality alone.

Secondary null hypotheses:

- None of the treatment modalities improves HRV as a measure of individual psychological stress in headache patients.
- None of the treatment modalities reduces the occurrence of tension-type headaches (days per month), the duration of pain episodes, or the consumption of analgesics by individuals.
- None of the treatment modalities influences health-related quality of life, physical and mental fitness, or psychological health.
- TMD as a comorbidity has no impact on treatment outcomes for tension-type headaches in the study setting.
- Kappa as a measure of inter-rater reliability of Chinese meridian diagnosis is <0.5 in comparisons.

## Methods/design

### Study design

The proposed study is a randomized controlled prospective trial with four balanced treatment arms. It will compare the efficacy of two distinct non-pharmacological treatment modalities (acupuncture vs. MTT), as monotherapies and in combination, with usual care according to IHS criteria for the treatment of common episodic and chronic tension-type headaches. Usual care is the control modality. Subjects will be assigned to one of the four treatment arms (usual care, acupuncture, MTT, and MTT plus acupuncture), using pre-generated randomization lists in a ratio of 1:1:1:1 with varying permutation block sizes. The randomization scheme was prepared by a scientific colleague who is not involved in scheduling, treatment, or evaluation. After the randomization list had been created, written allocation sheets were delivered to the treating physicians in sealed opaque envelopes.

The study is headed by the first and last authors as well as the head of the Departement of Rehabilitation medicine of the Hannover Medical School (CG). Protocol decisions were made in a peer process involving all co-authors. Changes to the study protocol can be made only with the written consent of the ethics committee.

### Sample size estimation

The sample size calculation was based on the results of Endres et al. [17]. The study was powered to detect a small interaction effect of time × intervention dose (*f* = 0.15) for the primary endpoint (α = 0.05) with a power of 80% in a variance analysis with five repeat measurements and four study groups. With these targets, 20 patients per study group were required for analysis. Assuming a dropout rate of 20% over the course of the trial, 24 patients must be included in each study group; thus, recruitment of 96 participants is required. The sample size calculation was performed using the G*Power 3 software.

### Patient recruitment

Patients will be invited to participate in the research study by notices in local and regional media and by calls for participation to staff members and students at the Hannover Medical School. Semi-structured telephone interviews and subsequent physical examinations will be performed to select patients with frequent episodic or chronic tension-type headaches according to the diagnostic criteria of the International Classification of Headache Disorders, 3rd Edition (IHS 2.2 and IHS 2.3).

After the telephone screening by two scientific assistants, a potential participant is registered and given an initial appointment, which includes a preliminary medical examination, checking of the inclusion and exclusion criteria, collecting comprehensive information, and providing consent to participate. After this appointment and consent to participate in the study, the subject draws one of the previously created opaque randomization envelopes, is assigned to the corresponding group, and is given dates for appointments for the intervention and for follow-up appointments. The appointments and schedules of the patients enrolled in the study are generated by a study nurse who is neither involved in the treatment nor in the randomization. Randomization will be overseen by one of the trial directors.

**Fig. 1:**
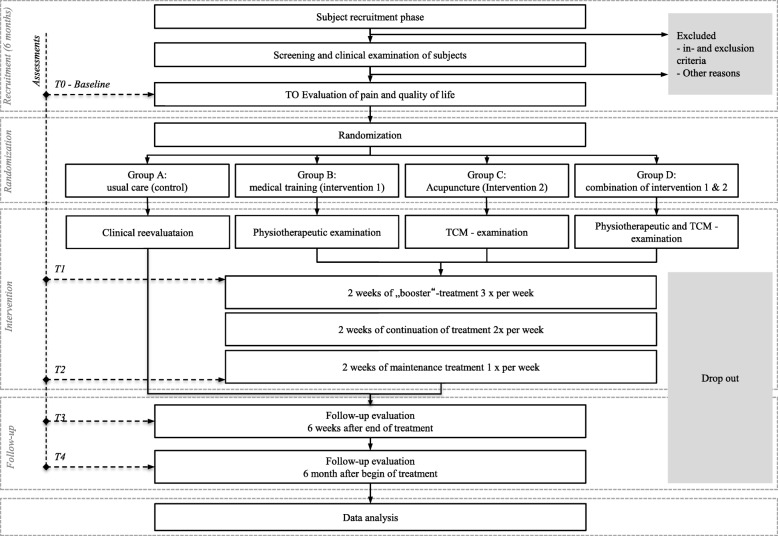
CONSORT flowchart [18]

**Fig. 2:**
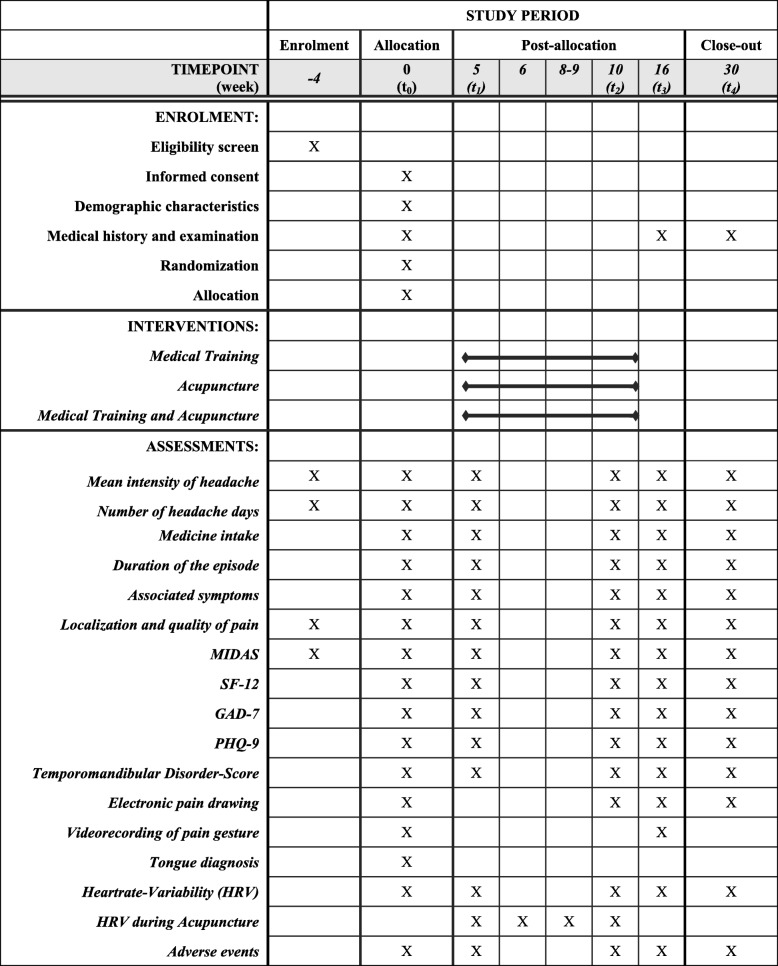
SPIRIT figure of enrolment, interventions, and assessments. GAD-7 Seven-item Generalized Anxiety Disorder Questionnaire, HRV Heart rate variability, MIDAS Migraine Disability Assessment, PHQ-D Patient Health Questionnaire, Depression, SF-12 12-item Short Form Health Survey

The total study period for each participant will be 7 months (Figs. 1 and 2). This period includes a 4-week baseline period, a 6-week intervention period, and a 20-week follow-up period. During the intervention period, the subjects in the trial groups will receive a total of 12 treatment units in decreasing frequency (three units per week during the first 2 weeks, two units per week during the third and fourth weeks, and one unit per week during the last 2 weeks). During the entire study period, as-needed analgesics (e.g. ibuprofen, aspirin, and paracetamol) can be taken as usual and will be documented in a headache diary.

### Inclusion criteria

To be eligible, patients must be aged between 18 and 65 years and have suffered from frequent episodic or chronic tension-type headaches for more than 6 months. An episode of headache must have occurred on at least 1 day per month for at least 3 months, the duration of which must be at least 30 min but no longer than 7 days continuously. Furthermore, subjects must be capable of fully completing all assessments.

### Exclusion criteria

Those who fulfill any of the following criteria will be excluded:

- Those participating or having participated during the last 30 days prior to the initial screening examination in another clinical trial
- Those who have been treated with acupuncture or MTT for tension-type headaches within the last 6 months prior to the initial examination
- Those with a serious psychiatric disorder
- Those with substance dependence or misuse
- Those using headache medication for >10 days per month
- Those with a severe neurological or severe medical disease
- Pregnant women
- Nursing women
- Women planning to become pregnant during the study period
- Those with migraine whose attacks occur more frequently than once per year
- Those with unstable angina pectoris
- Those with New York Heart Association functional classification III–IV heart failure
- Those with uncontrollable or untreatable arterial hypertension
- Those suffering from headaches due to other medical causes (e.g. pseudotumor cerebri, sleep apnea syndrome, or newly developed daily headaches)

### Dropout criteria

Subjects who refuse to undergo the assigned intervention and those who fail to complete the assessments will be excluded. The close patient contact due to the two or three intervention sessions per week is expected to promote patient adherence and minimize the dropout rate. In addition, we will personally remind the study participants by telephone before each follow-up appointment.

### Interventions

#### Acupuncture

The acupuncture treatment comprises a standardized combination of points plus additional individual points identified by location (meridian). Table 1 provides a detailed description of the acupuncture points used to allow replication of the intervention. All acupuncture points were selected based on TCM principles, the international literature, and the opinion of national and international experts. In line with the Standards for Reporting Interventions in Clinical Trials of Acupuncture criteria, the treatment was semi-standardized, fulfilling both the requirement of a basic individualized diagnosis and treatment and of a standardized acupuncture treatment for patients with comparable symptoms. In addition to these locations, needles will be inserted into 1–4 *ashi* points (i.e., points where the headache is most painful), if these points are clearly mentioned by the patient during the screening examination.

**Table 1:** Acupuncture treatment

Point selection	Pain localization	Acupuncture points	Depth [cun]
Standard		*Baihui* (GV20)	0.5–0.8
		*Taiyang* (EX-HN5)	0.3–0.5
		*Fengchi* (GB20)	0.8–1.2
		*Hegu* (LI4)	0.5–1.0
*Yangming* meridian	Front head, forehead, brow edge	*Neiting* (ST44)	0.5–0.8
		*Yintang* (GV29)	0.3–0.5
*Shaoyang* meridian	Side head, temporal	*Zulinqi* (GB41)	0.3–0.5
		*Waiguan* (SJ5)	0.5–1.0
*Taiyang* meridian	Back head, occipital	*Kunlun* (BL60)	0.5–1.0
		*Houxi* (SI3)	0.5–1.0
*Jueyin* meridian	Top of the head	*Taichong* (LR3)	0.5–0.8
		*Neiguan* (PC6)	0.5–1.0
		*Sishencong* (EX-HN1)	0.5–0.8

The detailed localizations, acupuncture point names, and insertion depths follow the rules of TCM. All acupuncture points will be manually stimulated on needle insertion and before removal of the needle. The *deqi* sensation will be evoked. The needle retention time will be 30 min. Sterile disposable acupuncture needles will be used (25–40 mm × 0.25–0.3 mm; manufacturer: Suzhou Tianxie Acupuncture Instruments Co, or similar). Acupuncture will be used without additional methods, such as moxibustion, cupping or electrical, stimulation, which extend the technique or treatment. Acupuncture will be performed by an experienced acupuncturist with more than 5 years of clinical experience in acupuncture treatment.

#### Medical training therapy

MTT will be provided by experienced and qualified physiotherapists. Based on an initial 45-min physiotherapeutic examination plus a cardiac stress test and testing of strength, endurance, posture control, and flexibility, a training plan will be created. The 12 standardized treatment units will have a duration of 60 min. The intensity will be tailored to the individual patient. A compact self-training program will be handed out, in writing, to the subject so that they can perform the exercises they have learned independently three times a week. Treatment generally adheres to the principles of MTT and comprises a combination of strength training, endurance training, flexibility training (yoga exercises), and posture training with coordinative elements. Table 2 provides a detailed description of the MTT approach used to allow replication of the training program.

**Table 2:** Medical training therapy

Method	Intervention	Intensity	Frequency	Duration
Cardiovascular training	Cycling	75% of maximum Heart Rate	1	15 min
	Rowing machine			
	Cross trainer			
	Treadmill			
Strength and endurance training	Pull-down	40% of max. strength	2 × 25	10
	Butterfly reverse			
	Rowing			
Coordinative training	Postural training	Individual level	1	3
Proprioceptive training	Segmental stabilization of the spine in flection and extension		2.5–4.8 Hz	5 × 15 s
	Segmental stabilization of the spine in lateral flexion			
	Reducing muscle tension			
Training to improve mobility and flexibility	Stretching the erector spinae muscles	Individual level	1	30 s
	Stretching the iliopsoas muscle and extending the flank		4 × 1	4 × 20 s
	Spinal mobilization from the quadruped stand		4 × 1	4 × 20 s
	While supine, extension and abdominal respiration, shoulder bridge and candle		2	30 s
	Forearm plank		2 × 1	30 s
	Rotational strain of the spine with breathing recess		5	5 × 10 s
	Stretching the trapezius and pectoralis muscles		2	30 s

#### Combination of acupuncture and MTT

The complex health intervention to be evaluated in the study is the combination of MTT and acupuncture. Following each MTT session and a short rest, the patients will receive acupuncture treatment.

#### Control group

Participants in the control group may continue to take as usual any IHS guideline-compliant existing symptomatic treatments with analgesics for acute attacks of headache on no more than 10 days per month. For those with chronic tension-type headaches, any pharmacological or non-pharmacological prophylactic treatment compliant with the guideline may also be continued (e.g. amitriptyline or relaxation techniques). After completing the last follow-up assessment 6 months after the initial screening examination, those in the control group will be offered the choice of acupuncture and MTT free of charge over a predefined period of 6 weeks.

### Outcome measurements

#### Primary outcome

To test the primary null hypothesis, the change in mean pain intensity (numerical rating scale) before the start of the intervention (T1) compared with the pain intensity 6 weeks after the end of the intervention (T3) is used as the primary endpoint.

#### Secondary outcomes

One of the secondary endpoints is the frequency of pain episodes in days per month, from which the number of responders (those with at least a 50% pain reduction) and the frequency of occurrence can be calculated.

Other secondary endpoints are the duration of headache attacks, the consumption of pain medication for tension-type headaches, health-related quality of life, effect of headaches on fitness and functional impairments (activities and participation), psychological health (mood, drive, anxiety, etc.), and autonomic factors (sleep–wake patterns). These outcomes will be measured using the Migraine Disability Assessment questionnaire [19], parts of the Patient Health Questionnaire for Depression (PHQ-D) [20], the nine-item Patient Health Questionnaire (PHQ-9) [21], the seven-item Generalized Anxiety Disorder (GAD-7) questionnaire [22], and the 12-item Short Form Health Survey (SF-12) [23]. Because anxiety, in particular, has a significant impact on the expectation of therapy, participants provide a brief written assessment of anxiety prior to each acupuncture treatment, in addition to the standardized assessments of anxiety and depression (PHQ-9 and GAD) -7 T0 to T4) - to evaluate treatment expectations. [24, 25].

At the visit to collect informed consent and at the first medical examination, acupuncture and MTT were presented as equally effective therapies for tension-type headaches to objectify their credibility and the expectations of patients [26]. To evaluate their expectations of the individual therapeutic procedures, they will complete a three-part survey after the intervention phase. On a scale of 0–10 (0 = does not apply, 10 = fully agrees), they will be asked to rate: (1) their expectation of the assigned therapy procedure, (2) whether that expectation was fulfilled, and (3) whether they would recommend the intervention to a relative or friend.

After receiving written and verbal instructions, the patients will independently complete the questionnaires. The completed questionnaires, including their study ID, will be given to the study nurse in a sealed envelope. The outcomes will be entered into a database by a member of the scientific staff who is not involved in the treatment. The data will then be reviewed by two of the principal investigators.

### Safety evaluation

All side effects that occur within the study period will be recorded. These will immediately be reviewed by the study physicians and will be considered in the evaluations. The study physicians will assess the relationship between the intervention and the severity of each adverse reaction. Independent safety monitoring will be provided by a researcher (CE) who is not involved in the study process and works with the university’s independent Medical Process and Patient Safety Unit. Suspected unexpected serious adverse reactions will be reported to the safety unit and the ethics committee. All study participants are covered throughout the trial by insurance. Due to the proven effectiveness of acupuncture and MTT, we consider that a loss of confidence by participants is unlikely. Patients may terminate their participation at any time for any reason. Participants may request a meeting with a study doctor to discuss an alternative treatment. To avoid dropouts, the participants will complete, at several time points, a survey on the organization, processes, possible problems, and their satisfaction with their interactions with the study staff.

### Measuring individual psychological stress levels

The effect of the interventions on individual psychological stress levels will be determined by measuring treatment-related changes in HRV. Using a wireless combination of a heart-rate belt and a tablet PC (VNS Analyse Professional, Commit GmbH, Liebenburg, Germany), a sitting electrocardiogram will be recorded after a short resting period. After automatic R wave identification, various HRV metrics will be determined. Of special interest are markers of parasympathetic regulation, such as the high-frequency component of HRV (0.15–0.4 Hz) and the root-mean square differences of successive R-R intervals, as well as markers of baroreflex regulation, such as the low-frequency component of HRV (0.04–0.15 Hz). Furthermore, by measuring HRV during acupuncture, distinct types of response to the stimulus can be explored and their potential influence on the efficacy of the treatment evaluated.

### Clinical examination for temporomandibular dysfunction

TMD will be diagnosed using the Research Diagnostic Criteria for Temporomandibular Disorders [27], which have been applied in many international studies. To ensure that the assessments are performed according to these criteria by a specialist, a research collaboration was established with the Department of Prosthetic Dentistry at the Hannover Medical School and a dentist was included in the study team (AW).

### Pain radiation

The precise headache pattern will be documented on a pain drawing created by the patient using an electronic pen on a tablet PC [28] running software SymptomMapper, which provides head outlines from four different views. Patients first choose descriptive terms for their pain and then color in all affected areas where they experience pain. Pain area and mean pain intensity will be calculated from these drawings.

### Pain gesture

Using a video recording of a patient’s pain body language (their pain gestures), their pain will be assessed before (T0) and 6 weeks after the intervention (T3). In addition to the location, which can be determined precisely by the pain gesture, the way the gesture is made will be discussed as a potential clinical sign in combination with the location. First, by analyzing the observed pain gestures, categories and pain clusters that may help in the diagnosis of tension-type headaches will be created according to the fascial distortion model described by Typaldos [29], because of the lack of previous studies. A fundamental aspect of how the cause of pain is identified in the fascial distortion model is through observing body language, which helps to give a precise differentiation of the symptoms. Then, these categories and pain clusters will be assessed for correlations with the TCM diagnoses.

### Data collection and management

All medical information will be documented and stored separately from personally identifiable data. The data will be recorded on the paper version of case report forms and double entered into a database by outcome assessors. The data sheets will be coded, and the group allocation will not be visible to third parties. Only pseudonymized data will be used for analysis and only members of the study group will be allowed to access these data to preserve patient confidentiality. The acupuncturist, physiotherapists, and statisticians will not have access to these data during patient evaluations. Independent data monitoring is performed by a scientist (JB) who is not involved in the study process. If subjects withdraw from the study, no further data will be collected from them. Data already obtained will be used in the intention-to-treat analysis with the consent of the patient or otherwise completely deleted from the database.

### Statistical analysis

Descriptive statistics will be used to describe the study population. The comparability of the study groups regarding age, gender, and outcome characteristics will be determined using an analysis of variation (ANOVA) or chi-squared tests. A significance level of *p* < 0.05 will be used for all tests. Analyses will be performed on an intention-to-treat basis. Multiple imputation will be used to handle missing data. To test the primary hypothesis, potential interaction effects (time × intervention) with pain as the primary endpoint will be calculated using a repeated-measures ANOVA. Supplementary Bonferroni-corrected post hoc tests will assess potential intragroup effects. Secondary interval-scale outcome measures will be analyzed using the same strategy. The proportion of treatment responders (those with at least a 50% reduction in the frequency of pain episodes) will be determined in supplementary analyses for the various treatment arms.

## Discussion

**Fig. 3:**
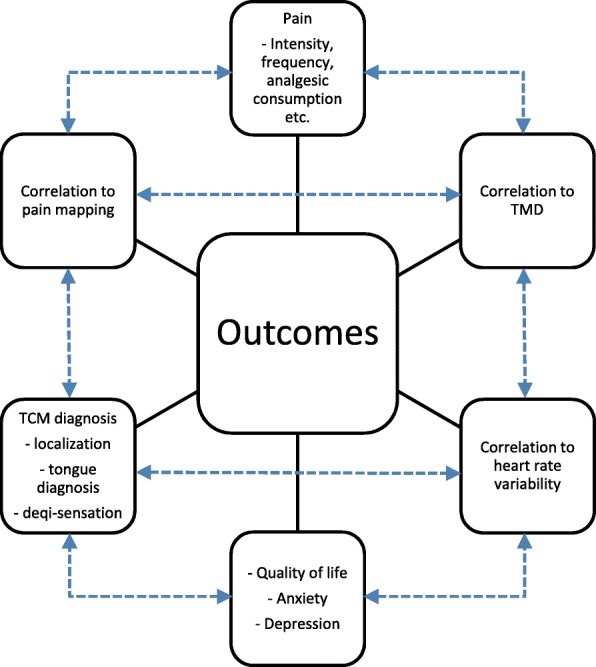
How the outcomes are related to each other. TCM traditional Chinese medicine

We expect to find positive treatment effects in all intervention groups for all outcomes in comparison with the usual care or control group. Furthermore, we expect to demonstrate the additive effects of acupuncture and MTT as a combination treatment and to provide scientific evidence to support the implementation of this complex health intervention. Since the two interventions differ in their mechanisms of action, consequently they exert analgesic, autonomic, and psychological effects at different levels. Thus, we expect different effects for each group for pain, the primary endpoint, and for the various secondary endpoints, such as mood, psychological stress, quality of life, and frequency of pain episodes. In addition, we believe that analyzing the correlations between the various outcome parameters with the applied treatment will provide information about the levels at which the effects occur. Furthermore, the influence of temporomandibular disorder on treatment effects will be a basic component of the data analysis. Figure 3 shows how the outcomes are related to each other. An evaluation of adverse events and their severity are important for safety and for patient acceptance of the treatment. Finally, these treatment modalities will be rated by the participating patients with regard to content and time requirements.

The innovative diagnostic tools (pain mapping and a video analysis of pain gestures) used in the study will provide further valuable outcomes due to their relevance and the additional information. A positive correlation between the patient’s pain mapping and the independent TCM diagnosis, which focuses on the meridians affected, will bring an improved and objective TCM diagnosis to everyday clinical practice. The same applies to pain gesture analysis, which has so far received insufficient attention, even though it broadens the possibilities for differential diagnoses of headaches and may have a decisive role by informing treatment decisions. A tongue diagnosis and its inter-rater reliability will also be addressed in the analysis.

A dedicated research outpatient clinic for integrative Chinese medicine was established for this study with the support of the China Academy of Chinese Medical Sciences. Thus, the proposed research project will also offer the opportunity to evaluate the feasibility of integrating such an outpatient clinic into the day-to-day operations of a medical university.

The main results for the primary outcomes are expected to be offered to the journal *Cephalalgia* as an alternative to the journal *Headache* for publication. The complementary aspects will be disseminated as follows. HRV results will be offered to *Autonomic Neuroscience*, TMD results to *Journal of Oral Rehabilitation*, and pain body gesture results to *Complementary and Alternative Medicine*. Oral and poster presentations will be made in international congresses. Extracts from the results of the study will be made accessible to the public, after their publication in journals, on the homepage of the clinic and in newsletters, as well as the print and online media of the Hannover Medical School press office (for example MHH Info, research reports, and information for patients and doctors).

### Limitations

The following four factors are potential confounders and concomitant variables:

1. Changes in individual behavior during the study. These could result from contact with the therapists or from directing attention to the symptoms. Behavioral changes will, as far as possible, be recorded in a behavior diary.
2. Changes in other therapies, including self-treatment. These can include movement therapies or the intake of over-the-counter medication. Such changes will also be recorded in diaries if possible, to enable a correction of the results where necessary.
3. Unspecific treatment effects. These can differ between the various study arms. For example, depending on treatment expectations, the unspecific effects of acupuncture can be stronger than those of physical training. In addition, there may be cultural influences and treatment expectations. These cannot be individually determined because of the excessive effort that would involve; however, potential effects will be addressed in the discussion of the results.
4. Seasonal influences on pain. Such are likely to be present, but cannot be accounted for in the study. However, since the various study arms will be evaluated in parallel, potential effects would affect all study groups equally.

### Trial status

The trial commenced at the beginning of 2018. The approval of the ethics committee was given in June 2018. The recruitment of participants began in July 2018 and was completed in June 2019. The study will be finished in January 2020.

## Supplementary information

**Additional file 1.** SPIRIT checklist.

## Data Availability

Not applicable

## Abbreviations

ANOVA: Analysis of variance
GAD-7: Seven-item Generalized Anxiety Disorder Questionnaire
HRV: Heart rate variability
IHS: International Headache Society
MIDAS: Migraine Disability Assessment
MTT: Medical training therapy
PHQ-9: Nine-item Patient Health Questionnaire
PHQ-D: Patient Health Questionnaire, Depression
SF-12: 12-item Short Form Health Survey
SPIRIT: Standard Protocol Items: Recommendations for Intervention Trials
TCM: Traditional Chinese medicine
TMD: Temporomandibular disorder

## References

[1] Linde K, Allais G, Brinkhaus B, Mannheimer E, Vickers A, White AR (2016). Acupuncture for the prevention of tension-type headache. Cochrane Database Syst Rev.

[2] Gil Martinez A, Kindelan P, Agudo-Carmona D, Muñoz Plata R, López-de-Uralde-Villanueva I, La Touche R (2013). Therapeutic exercise as treatment for migraine and tension-type headaches: a systematic review of randomised clinical trials. Rev Neurol.

[3] Melchart D, Streng A, Hoppe A, Brinkhaus B, Witt C, Wagenpfeil S (2005). Acupuncture in patients with tension-type headache: randomised controlled trial. Bmj..

[4] Jena S, Witt CM, Brinkhaus B, Wegscheider K, Willich SN (2008). Acupuncture in patients with headache. Cephalalgia..

[5] Vickers AJ, Cronin AM, Maschino AC, Lewith G, MacPherson H, Foster NE (2012). Acupuncture for chronic pain: individual patient data meta-analysis. Arch Intern Med.

[6] Torelli P, Jensen R, Olesen J (2004). Physiotherapy for tension-type headache: a controlled study. Cephalalgia..

[7] Hammill JM, Cook TM, Rosecrance JC (1996). Effectiveness of a physical therapy regimen in the treatment of tension-type headache. Headache..

[8] Söderberg E, Carlsson J, Stener-Victorin E (2006). Chronic tension-type headache treated with acupuncture, physical training and relaxation training. Between-group differences. Cephalalgia..

[9] Carlsson J, Fahlcrantz A, Augustinsson LE (1990). Muscle tenderness in tension headache treated with acupuncture or physiotherapy. Cephalalgia..

[10] Edmeads J, Findlay H, Tugwell P, Pryse-Phillips W, Nelson RF, Murray TJ (1993). Impact of migraine and tension-type headache on life-style, consulting behaviour, and medication use: a Canadian population survey. Can J Neurol Sci.

[11] Rasmussen BK, Jensen R, Olesen J (1992). Impact of headache on sickness absence and utilisation of medical services: a Danish population study. J Epidemiol Community Health.

[12] Speciali JG, Dach F (2015). Temporomandibular dysfunction and headache disorder. Headache..

[13] Lim PF, Smith S, Bhalang K, Slade GD, Maixner W (2010). Development of temporomandibular disorders is associated with greater bodily pain experience. Clin J Pain.

[14] Straube A, Diener HC, Weimar C (2012). Therapie des episodischen und chronischen Kopfschmerzes vom Spannungstyp und anderer chronischer täglicher Kopfschmerzen. Entwicklungsstufe: S1. Kommission “Leitlinien” der Deutschen Gesellschaft für Neurologie.

[15] International Headache Society (2018). Headache Classification Committee of the International Headache Society (IHS). The international classification of headache disorders, 3rd edition. Cephalalgia..

[16] Godlee F (2018). Acupuncture: theatrical placebo or caring approach to pain?. BMJ.

[17] Endres HG, Böwing G, Diener HC, Lange S, Maier C, Molsberger A (2007). Acupuncture for tension-type headache: a multicentre, sham-controlled, patient-and observer-blinded, randomised trial. J Headache Pain.

[18] Cochrane Database of Systematic Reviews (2010). Consolidated standards of reporting trials (CONSORT) and the completeness of reporting of randomised controlled trials published in medical journals. Review content assessed as up-to-date.

[19] Stewart WF, Lipton RB, Dowson AJ, Sawyer J (2001). Development and testing of the Migraine Disability Assessment (MIDAS) Questionnaire to assess headache-related disability. Neurology..

[20] Gräfe K, Zipfel S, Herzog W, Löwe B (2004). Screening psychischer Störungen mit dem “Gesundheitsfragebogen für Patienten (PHQ-D)”. Ergebnisse der deutschen Validierungsstudie. Diagnostica..

[21] Kroenke K, Spitzer RL, Williams JB (2001). The PHQ-9: validity of a brief depression severity measure. J Gen Intern Med.

[22] Löwe B, Decker O, Müller S, Brähler E, Schellberg D, Herzog W (2008). Validation and standardization of the Generalized Anxiety Disorder Screener (GAD-7) in the general population. Med Care.

[23] Ware J, Kosinski M, Keller SD (1996). A 12-Item Short-Form Health Survey: construction of scales and preliminary tests of reliability and validity. Med Care.

[24] Valença MM (2016). Commentary: Acute Tension-Type Headaches Are Associated with Impaired Cognitive Function and More Negative Mood. Front Neurol.

[25] Song TJ, Cho SJ, Kim WJ, Yang KI, Yun CH, Chu MK (2016). Anxiety and Depression in Tension-Type Headache: A Population-Based Study. PLoS One.

[26] Sacristán JA, Aguarón A, Avendaño-Solá C, Garrido P, Carrión J, Gutiérrez A (2016). Patient involvement in clinical research: why, when, and how. Patient Prefer Adherence.

[27] Dworkin SF, LeResche L (1992). Research diagnostic criteria for temporomandibular disorders: review, criteria, examinations and specifications, critique. J Craniomandib Disord.

[28] Neubert TA, Dusch M, Karst M, Beissner F (2018). Designing a tablet-based software app for mapping bodily symptoms: usability evaluation and reproducibility analysis. JMIR mHealth and uHealth.

[29] Typaldos S (1994). Introducing the fascial distortion model. AAO J.

